# Lyoprotectant Constituents Suited for Lyophilization and Reconstitution of Stem-Cell-Derived Extracellular Vesicles

**DOI:** 10.34133/bmr.0005

**Published:** 2024-02-02

**Authors:** Wu Young Kang, Eun Kyoung Shin, Eun Hee Kim, Min-Ho Kang, Chi Young Bang, Oh Young Bang, Jae Min Cha

**Affiliations:** ^1^ Department of Biomedical & Robotics Engineering, College of Engineering, Incheon National University, Incheon 22012, Republic of Korea.; ^2^ 3D Stem Cell Bioengineering Laboratory, Research Institute for Engineering and Technology, Incheon National University, Incheon 22012, Republic of Korea.; ^3^ S&E bio Co., Ltd., Seoul 06351, Republic of Korea.; ^4^ Department of BioMedical-Chemical Engineering (BMCE), The Catholic University of Korea, Bucheon 14662, Republic of Korea.; ^5^ Department of Biotechnology, The Catholic University of Korea, Bucheon 14662, Republic of Korea.; ^6^ Department of Plastic and Reconstructive Surgery, Kangwon National University Hospital, Chuncheon 24341, Republic of Korea.; ^7^ Department of Neurology, Samsung Medical Center, Sungkyunkwan University School of Medicine, Seoul 06351, Republic of Korea.

## Abstract

Stem-cell-derived extracellular vesicles (EVs) are emerging as an alternative approach to stem cell therapy. Successful lyophilization of EVs could enable convenient storage and distribution of EV medicinal products at room temperature for long periods, thus considerably increasing the accessibility of EV therapeutics to patients. In this study, we aimed to identify an appropriate lyoprotectant composition for the lyophilization and reconstitution of stem-cell-derived EVs. MSC-derived EVs were lyophilized using different lyoprotectants, such as dimethyl sulfoxide, mannitol, trehalose, and sucrose, at varying concentrations. Our results revealed that a mixture of trehalose and sucrose at high concentrations could support the formation of amorphous ice by enriching the amorphous phase of the solution, which successfully inhibited the acceleration of buffer component crystallization during lyophilization. Lyophilized and reconstituted EVs were thoroughly evaluated for concentration and size, morphology, and protein and RNA content. The therapeutic effects of the reconstituted EVs were examined using a tube formation assay with human umbilical vein endothelial cells. After rehydration of the lyophilized EVs, most of their generic characteristics were well-maintained, and their therapeutic capacity recovered to levels similar to those of freshly collected EVs. The concentrations and morphologies of the lyophilized EVs were similar to the initial features of the fresh EV group until day 30 at room temperature, although their therapeutic capacity appeared to decrease after 7 days. Our study suggests an appropriate composition of lyoprotectants, particularly for EV lyophilization, which could encourage the applications of stem-cell-derived EV therapeutics in the health industry.

## Introduction

Extracellular vesicles (EVs) such as exosomes and microvesicles derived from stem cells contain multiple therapeutic factors including various proteomic and/or genomic compounds secreted from stem cells [1,2]. Such therapeutic molecules in EVs are securely encapsulated by a phospholipid bilayer, as nano-sized vesicles typically range between 30 and 1,000 nm, which are known to be beneficial in systemic circulation [3–5]. In addition, the lipid membranes of EVs are embedded with many receptors present in the stem cell membrane; therefore, EVs are capable of homing to the pathological environment, similar to their parental cells [6]. Since stem cell therapies possess critical concerns in the consistency of therapeutic efficacy and safety issues as living stem cells are injected into a human body, stem-cell-derived EV therapy is emerging as a new paradigm to more efficiently apply stem cells’ therapeutic effects to clinics [7]. In addition, EV therapeutics exhibit many benefits concerning biocompatibility, immunogenicity, stability, pharmacokinetics, and biodistribution, compared to current stem cell therapies [8].

Despite the obvious advantages, developing technology to preserve viable EVs for long periods at room temperature while securing stable biological formulations is still insufficient. When stored at room temperature, stem-cell-derived EVs containing protein- and/or nucleic-acid-based therapeutic factors can decompose, affecting the expected therapeutic effects [9]. It has been reported that even when refrigerated, the generic characteristics of EVs, including numbers, sizes, morphologies, and protein and RNA contents, progressively change with storage time, and the activities of the major therapeutic molecules decline within a few days [10]. Freezing at −80 °C could preserve the properties of EVs for a relatively long period, which is most widely used for storing EVs [11]. However, it has been recently revealed that Pfizer and Moderna’s mRNA vaccines have many inconvenient storage and distribution issues, such as requiring advanced cold chains below −80 °C to maintain their stability, which would cause further complications in developing countries or climatically unfavorable regions [12].

Lyophilization, also known as freeze-drying, is a cost-effective method for preserving labile substances. Lyophilization is particularly beneficial in the pharmaceutical industry because a water-containing drug product can be transformed into a stable dried solid or powder form by sublimation, and therapeutic formulations can be efficiently secured, easily packaged, and distributed as the final drug product [13]. Lyophilized drug products can also be conveniently reconstituted by healthcare professionals at the bedside immediately before administration to patients. Developing a lyophilized formulation of EVs could significantly extend the accessibility of patients to the benefits of EV therapeutics as it enables long-term preservation at room temperature and allow convenient and cost-effective shipping and distribution.

Successful lyophilization of biological samples such as EVs relies considerably on using an appropriate lyoprotectant. Many previous studies have utilized various types of disaccharides, such as trehalose and sucrose, as lyoprotectant compositions to increase viscosity and prevent ice crystallization by forming a glassy state, which, in turn, supports the formation of amorphous ice during the freezing process [14]. During the drying process, the lyoprotectant solution becomes glassier with increasing concentration so that the movement of non-covalently bonded chains of biomolecules is disallowed, preserving the original architecture and physicochemical properties of the biological samples [15]. In addition, sugar molecules retain a large number of hydroxyl groups that can form abundant hydrogen bonds with the surface molecules of the lipid layers in biological samples [16]. When the sublimation of water in the drying process causes membrane destabilization of biological samples because of the loss of hydrogen bonds between lipid layers, the sugar molecules in the lyoprotectant can protect the samples from dehydration-induced shrinkage by replacing water molecules [17].

**Fig. 1.**
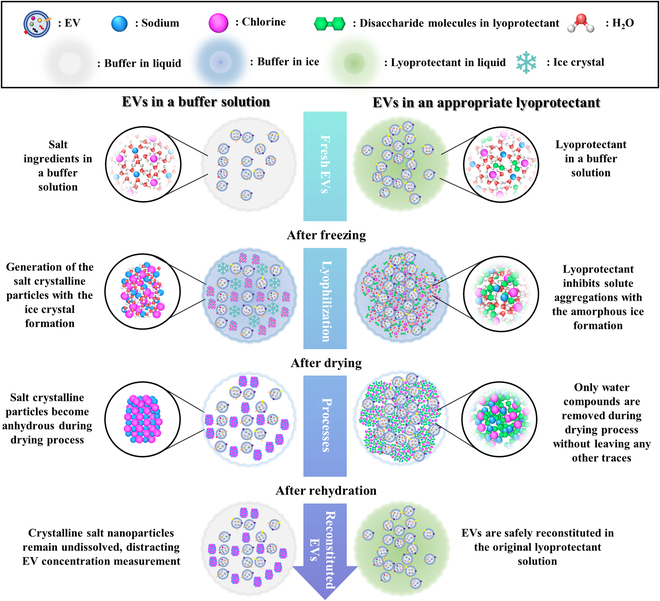
Schematic diagram of the presence or absence of the lyoprotectant during lyophilization process.

This study investigated different lyoprotectants such as dimethyl sulfoxide (DMSO), mannitol, trehalose, and sucrose with varying concentrations for EV lyophilization and long-term preservation at room temperature. Based on these results, a mixed combination of trehalose and sucrose at high concentrations is suggested as an appropriate composition of lyoprotectants for EVs, and critical issues related to EV lyophilization were discussed (Fig. 1).

## Materials and Methods

### Cell culture

#### Human bone-marrow-derived mesenchymal stem cells

Human bone-marrow-derived mesenchymal stem cells (BM-hMSC; Lonza, Basel, Switzerland) were cultured in 100-mm culture dishes (Corning, Tewsburg, MA, USA) in Dulbecco’s modified eagle’s medium with low glucose (DMEM; Invitrogen, Carlsbad, CA, USA) supplemented with 10% fetal bovine serum (FBS; Invitrogen) and 1% antibiotic–antimycotic (anti–anti; Invitrogen). MSCs were detached with TrypLE (Life Technologies, Carlsbad, CA, USA) at 70% confluency, and seeded in 100-mm culture dishes at 4,500 cells/cm^2^ until passage 6. To collect EVs for analysis, cells were cultured for 5 days in DMEM supplemented with 10% exosome-depleted FBS (exo-FBS; System Biosciences, Palo Alto, CA, USA) and 1% anti–anti. Cells were incubated at 37 °C and 5% CO_2_.

#### Human umbilical cord Warton’s jelly-derived mesenchymal stem cells

Umbilical cords obtained from normal pregnancy from 3 donors were used. Umbilical cord Warton’s jelly (WJ) tissue samples underwent multiple washes with phosphate-buffered saline (PBS; Life Technologies) and were mechanically minced using surgical scissors. Tissue digestion was carried out using 0.1% collagenase type I solubilized in PBS at 37 °C. After 1 h, MEM-alpha Minimum Essential Medium (Life Technologies) supplemented with 15% FBS was added to the cell suspension and then centrifuged at 600 × *g* for 5 min. MSCs were frozen by passage 4 and thawed using MEM-alpha supplemented with 10% FBS and 0.1% gentamicin (Life Technologies) until passage 6 for EV harvest. To collect EVs for analysis, cells were cultured until 80% confluency and the culture medium was changed to serum-free MEM-alpha with 0.1% gentamicin for 48 h. Cells were incubated at 37 °C and 5% CO_2_.

### Isolation of EVs using tangential flow filtration

The culture medium was harvested and centrifuged at 2,500 × *g* for 10 min to eliminate large cell debris. Then, the supernatant was filtered using bottle top filters (Life Technologies) with 0.2 μm polyethersulfone membrane and purified by a tangential flow filtration (TFF) system using a 300-kDa cutoff TFF cartridge (Minimate; Pall Corporation, Port Washington, NY, USA). The medium was concentrated approximately 20-fold, and the buffer was replaced with PBS (Cytiva, Marlborough, USA). The harvested EVs were filtered by a 0.2-μm polyethersulfone membrane and stored or mixed with lyoprotectant.

### EV size and concentration measurement using tunable resistive pulse sensing

A tunable resistive pulse sensing (TRPS) method using qNano (Izon Science, Christchurch, Canterbury, New Zealand) was used to measure the size and concentration of EVs. Before measurement, lyophilized EV groups were prepared at 5 to 20× dilution with an electric liquid (EL), mixed with PBS and coating powder (Izon Science), and then EVs were placed above the nanopore and the EL was placed below. For each experiment, the stretching of the nanopores and the voltage were fine-tuned to measure the particles in the detection range. In addition, each sample was calibrated using a CPC100 (Izon Science) with a mean size of 100 nm.

### EV lyophilization and rehydration

EVs were lyophilized using trehalose (T; Sigma-Aldrich, St. Louis, MO, USA), sucrose (Sigma), mannitol (M; Sigma), and DMSO (Sigma). Each lyoprotectant solution was prepared at a 2-fold concentration in the experimental group and mixed with 2 × 10^11^ particles/ml of EVs in a 1:1 ratio to obtain 1 × 10^11^ EVs in a 1-ml vial. A trehalose and sucrose mixture (TS) was mixed with the EVs similarly. The resulting solutions were frozen at −80 °C overnight, then dried at −80 °C, further dried at 5 mTorr for 4 days on a tabletop freeze-dryer (ilShinBioBase, Gyeonggi-do, Korea), and stored in a room-temperature desiccator (Korea Ace Scientific, Seoul, Korea) until use. The lyophilized EVs were rehydrated by adding deionized (DI) water based on the volume before lyophilization at 37 °C. The same method was used for experiments without EVs.

### Evaluating crystallinity using x-ray diffraction

To evaluate the crystallinity of the lyoprotectant solutions, each experimental composition was exposed to Cu radiation (45 kV × 200 mA) using x-ray diffraction (XRD) SmartLab (Rigaku Corporation, Tokyo, Japan). The range of 2θ was 2° to 45°, the step size was 0.05°, and the dwell time was 2 s.

### Measuring glass transition temperature using differential scanning calorimetry

The glass transition temperature (Tg′) of the freeze-concentrated solution was measured using a NEXTA DSC200 (HITACHI, Tokyo, Japan). Approximately 7.5 μl of each lyoprotectant solution in an aluminum pan (GCA-0052; HITACHI) was cooled down to −60 °C and heated at a rate of 1 °C/min to 10 °C and compared to an empty aluminum pan as a control. The Tg of each group was estimated as the midpoint of the transition.

### Scanning electron microscopy and energy-dispersive x-ray spectroscopy

The morphology of each lyophilized composition was investigated using scanning electron microscopy (SEM; JSM-7800F; JEOL, Tokyo, Japan). Each group of lyophilized powder was fixed to a carbon tape and then subjected to platinum coating. It was performed while controlling the power from 5 to 15 kV in the range without damage to the samples. Additionally, energy-dispersive x-ray spectroscopy (EDS) was performed to identify the properties of the particles, showing the color mapping of carbon (red), oxygen (yellow), sodium (blue), and chlorine (pink) including overlapped images.

### Quantification of proteomic and genomic contents of EVs

All rehydrated EVs were centrifuged for 1 h at 120,000 × *g* in an ultracentrifuge (Beckman Coulter, Brea, CA, USA), and the supernatant was discarded. The pellet for protein quantification was lysed with radioimmunoprecipitation assay buffer (RIPA buffer; Life Technologies), and RNA was performed using TRIzol (Invitrogen). To quantify the amounts of protein, a bicinchoninic acid (BCA; Invitrogen) protein assay was used according to the manufacturer’s protocol, and the measured value was calibrated using a bovine serum albumin (BSA; Sigma) standard. In the case of RNA compounds, after the pellet was completely dissolved, total RNA was obtained from interphase separated by chloroform (Sigma), and washed once with isopropanol (Merck Millipore, Burlington, MA, USA), and twice with 70% ethanol (Merck Millipore). The ethanol was then removed and replaced with DI water, and the amount of RNA was measured using a Nanodrop 2000 (Life Technologies).

### Enzyme-linked immunosorbent assay

Enzyme-linked immunosorbent assay (ELISA) was performed using a commercial kit according to the manufacturer’s instructions. The CD63 (EH95RB, Life Technologies, Waltham, MA, USA) ELISA kit includes a standard protein; therefore, the amounts of protein and EVs were determined based on the kit’s standard curve. The isolated fresh EV and lyophilized EV were dispensed in equal amounts (200 μl/well) along with the standard into a capture antibody-coated 96-well microplate without pretreatment and then incubated at 4 °C. The next day, according to the Sandwich ELISA method, absorbance was measured using a microplate reader.

### Cryo-transition electron microscopy

Grids (Quantifoil, R 1.2/1.3, 200 mesh, EMS) were made on a hydrophilic surface with the glow discharge system (PELCO easiGlow; Ted Pella, Redding, CA, USA). Samples (3 or 4 μl) were added on the grid and blotted for 4 s at 100% humidity and a temperature of 15 °C or blotted for 1.5 s at 100% humidity and a temperature of 4 °C. The samples were then plunge-frozen for vitrification using a Vitrobot Mark IV (FEI Company, Hillsboro, Oregon, USA) in liquid ethane. The samples were analyzed using a Talos L120C (FEI Company) at 120 kV at the Nanobioimaging Center (Seoul National University, Korea) or a Glacios (Life Technology).

### Tube formation assay

The vascular regeneration ability of WJ-MSC-derived EVs was confirmed through a tube formation assay using human umbilical vein endothelial cells (HUVECs) [18,19]. More specifically, to see the angiogenesis effect of the EVs, the tube formation effect was confirmed by treating HUVECs attached to Matrigel (BD Bioscience, Franklin Lakes, NJ, USA) with EVs. HUVECs were cultured in M199 medium (Invitrogen) supplemented with 20% FBS, heparin (5 U/ml), and basic fibroblast growth factor (3 ng/ml). Cells were inoculated in growth-factor-reduced Matrigel Matrix from μ-Slides Angiogenesis (ibidi, Bavaria, Germany) at a density of 1.0 × 10^4^ cells and incubated in a chamber at 37 °C and 5% CO_2_ for 7 h for tube formation. Images were taken using a phase-contrast microscope (Olympus, Tokyo, Japan), and the number of tubular structures was quantified in the microscopic field (4× magnification) using ImageJ software.

### Statistical analysis

The results are presented as means ± standard deviations (SDs). A one-way analysis of variance (ANOVA) with Tukey’s post-hoc test was used. The number of iterations for each experiment is shown in the corresponding captions. *P < 0.05 and **P < 0.001 were used to indicate significance.

## Results

**Fig. 2.**
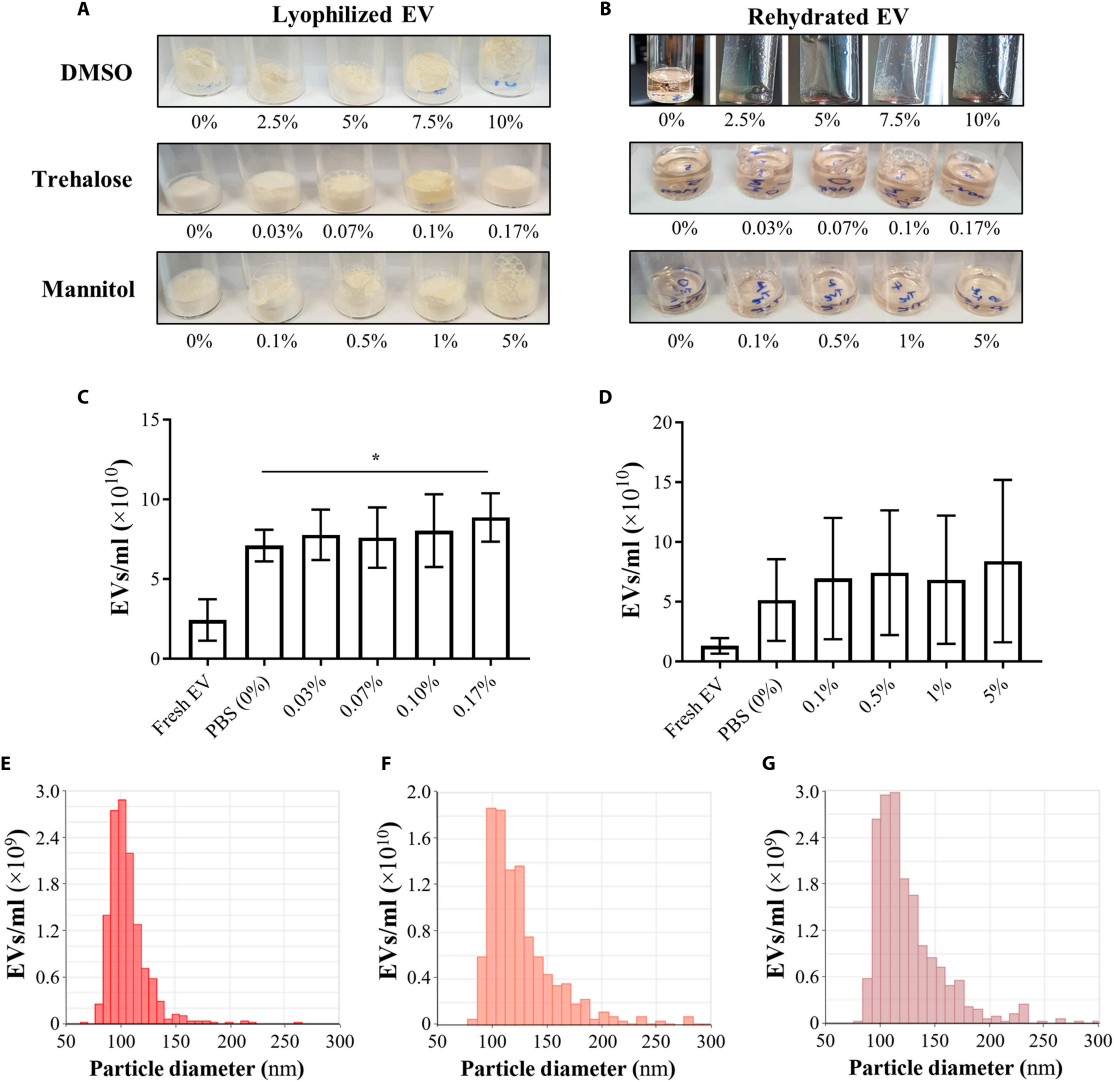
Evaluation of lyophilized EVs after rehydration. EVs were mixed with each lyoprotectant composition and finally prepared to 1 × 10^11^ EVs/ml and lyophilized. (A) Different cake appearances of lyophilized EVs using DMSO, trehalose, and mannitol and (B) appearances of them after rehydration. Particle concentration of EVs lyophilized with (C) trehalose and (D) mannitol compared to fresh EV (*n* = 3). Number-based particle size distribution of (E) fresh EV and EVs lyophilized in (F) 0.17% trehalose and (G) 5% mannitol solutions. Statistics was calculated using one-way ANOVA. *P < 0.05.

EVs derived from MSCs were lyophilized using different lyoprotectants at varying concentrations as previously reported (Fig. 2A): DMSO (0% to 10% v/v), trehalose (0% to 0.17% w/v), and mannitol (0% to 5% w/v) [20–22]. Lyophilization using a DMSO solution resulted in the formation of clumps that gathered as greasy films. After being dissolved in DI water, they were barely rehydrated as insoluble matter; thus, further nanoparticle measurements were not possible. Lyophilization with trehalose and mannitol solutions resulted in the formation of lyophilized cakes as fine-grained powders. After being dissolved in DI water, they tended to be rehydrated without forming insoluble matter during visual observation, unlike in the DMSO solutions. However, the results of the nanoparticle measurement of the rehydrated solutions revealed that their measured numbers were increased by about 8-fold or 9-fold higher than those of the fresh EV (each sample initially contained the same number of EVs), suggesting that there were some undesired nanoparticles with a similar size range to the EVs, generated during the lyophilization and/or rehydration processes (Fig. 2C to G).

**Fig. 3.**
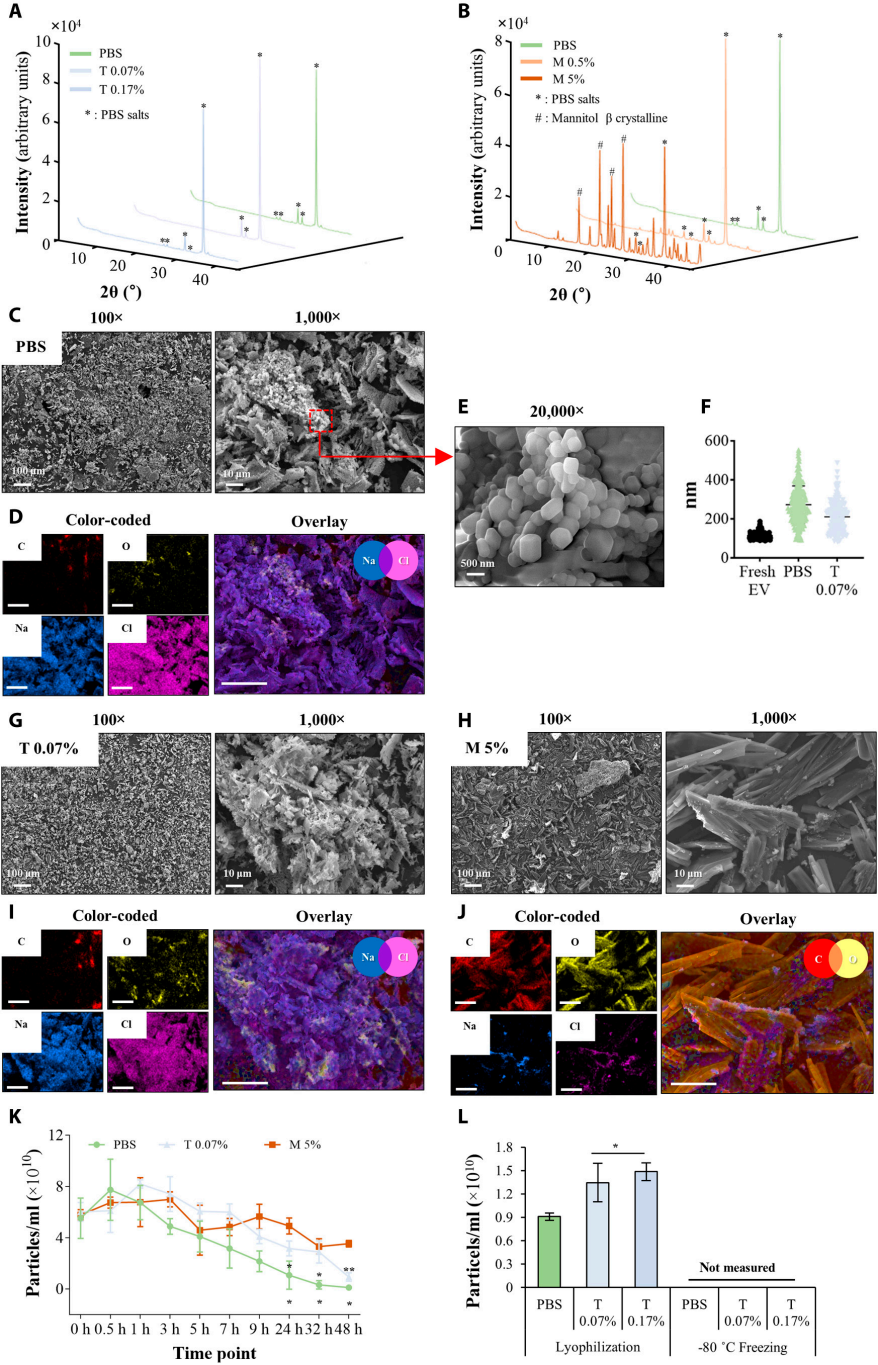
Identification and evaluation of lyophilized trehalose and mannitol solution (without EVs) to confirm the unidentified nanoparticles formed during lyophilization. XRD patterns of lyophilized (A) 0.07% and 0.17% trehalose and (B) 0.5% and 5% mannitol. The characteristic peaks of PBS salts (*) and mannitol β crystalline form (#) are identified. (C) Scanning electron microscopy (SEM) photographs including 100× and 1,000× images of PBS. (D) Energy dispersive x-ray spectroscopy (EDS) color-coded elemental mapping of 1,000× PBS images, indicating PBS salt component (purple): carbon (C; red), oxygen (O; yellow), sodium (Na; blue), and chlorine (Cl; pink). The scale bar indicates 25 μm. (E) High-magnification images of PBS and (F) comparison of mean size between fresh EV and PBS salt crystal formed in PBS and trehalose (T) 0.07%. It was performed by ImageJ (*n* = 6). SEM photographs of (G) 0.07% trehalose and (H) 5% mannitol. EDS elemental mapping of (I) 0.07% trehalose and (J) 5% mannitol, including PBS salt component (purple) and lyoprotectant component (orange). The scale bar indicates 25 μm. (K) Rehydration potential of lyophilized PBS, 0.07% trehalose, and 5% mannitol over time (*n* = 3). Significance was analyzed compared to 0 h. (L) Particle concentration of frozen and lyophilized trehalose (0.07% and 0.17%) (*n* = 3). *P < 0.05 and **P < 0.001 were calculated by one-way ANOVA compared to the PBS group. XRD, x-ray diffraction.

Therefore, we lyophilized and rehydrated PBS, mannitol (0.5% and 5%), and trehalose (0.07% and 0.17%) solutions without EVs and performed XRD analysis to identify the actual compositions of the unidentified nanoparticles (Fig. 3A and B). Undissolved PBS salts, characterized by peaks at 22° to 24°, 26° to 28°, and 31° to 33°, were detected in the rehydrated solutions of all groups (Fig. 3A) [23,24]. In particular, strong peaks of the mannitol β crystalline form at 14° to 15°, 16.8°, and 24.6° were detected in the samples lyophilized with mannitol (Fig. 3B), hardly redissolving once formed [24]. Undesirably, the nanoparticles obtained in the groups treated with PBS, trehalose at low concentrations (0.07%), and 5% mannitol were observed by SEM imaging (Fig. 3C, E, G, and H). The sizes of the nanoparticles in the SEM images overlapped with the mean size of fresh EVs (Fig. 3F). In addition, in the cases of PBS and 0.07% trehalose, the EDS elemental mapping results revealed that most of the nanoparticles were composed of sodium (blue) and chlorine (pink), which are the main components of the PBS salts, shown in purple (Fig. 3D and I). In particular, the formation of crystalline mannitol structures corresponds with the XRD patterns (Fig. 3J). The newly generated nanoparticles in the 0.07% trehalose solution, which were identified as undissolved aggregations of PBS salt crystals, and the 5% mannitol solution, which includes PBS salt crystals and the mannitol β crystalline form, remained in the DI water for up to 48 h (Fig. 3K). To investigate if the nanoparticles or undissolved PBS salt particles were generated during the pre-freezing step at -80 °C of the lyophilization process, nanoparticle measurement was conducted with PBS and trehalose solutions were frozen at −80 °C and thawed at room temperature (Fig. 3L). In contrast to the lyophilized and rehydrated groups, newly generated nanoparticles were not found in the frozen and thawed groups, which did not include the drying process.

**Fig. 4.**
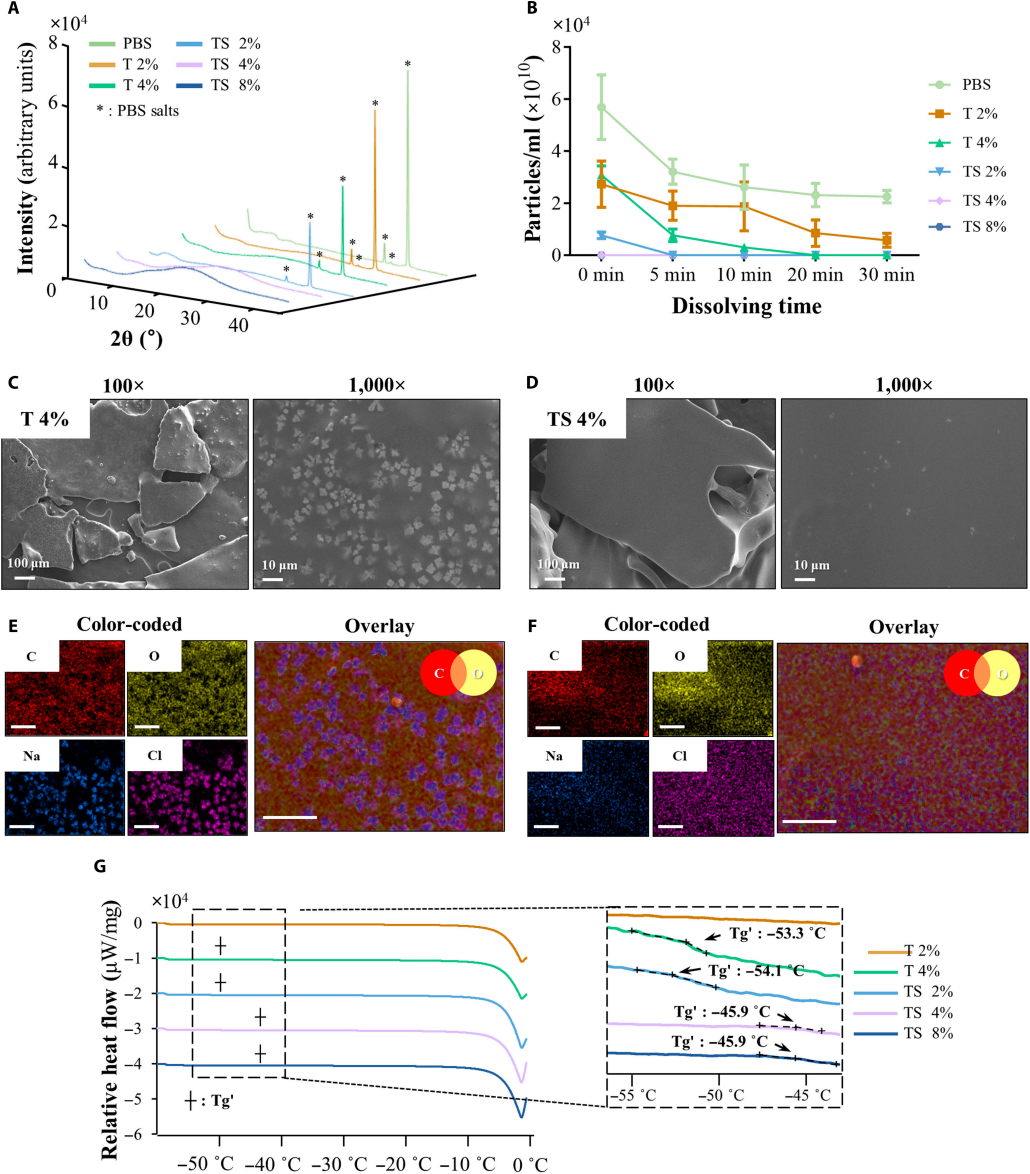
Evaluation of the new lyoprotectant compositions. (A) The characteristic XRD patterns of PBS salt in each lyophilized group. (B) Evaluation of the rehydration capacity at a time interval of 0 to 30 min using each composition: PBS, 2%, and 4% trehalose (T), trehalose 2% + sucrose 2% (TS 2%), trehalose 4% + sucrose 4% (TS 4%), and trehalose 8% + sucrose 8% (TS 8%) (*n* = 3). SEM images of (C) T 4% and (D) TS 4%. Color-coded EDS mapping of (E) T 4% and (F) TS 4%, including lyoprotectant component (orange). The scale bar of EDS indicates 25 μm. (G) DSC thermograms of each composition indicated glass transition. XRD, x-ray diffraction; DSC, differential scanning calorimetry.

We investigated higher concentrations, specifically 2% and 4% of trehalose (groups named “T 2%” and “T 4%”, respectively), as well as mixtures of 2% trehalose and 2% sucrose, 4% trehalose and 4% sucrose, and 8% trehalose and 8% sucrose (groups named “TS 2%”, “TS 4%”, and “TS 8%”, respectively) to study their effects on PBS salt formation during the lyophilization process. The formation of the PBS crystalline salt during lyophilization in each group was evaluated by XRD analysis (Fig. 4A). The results showed that the intensities of the peaks at 22° to 24°, 26° to 28°, and 31° to 33°, corresponding to the PBS salts, tended to decrease as the trehalose concentrations increased. In particular, the peaks of the PBS salts were hardly found in the TS 4% and 8% group, a mixture of trehalose and sucrose at higher concentrations. Nanoparticle measurement revealed that after 30 min of rehydration, nanoparticles were barely detected in the T 4%, TS 2%, 4%, and 8% (Fig. 4B). In particular, the nanoparticles in TS 2% were fully dissolved 5 min after rehydration, while TS 4% and 8% groups were done shortly after rehydration. These results (Fig. 4A and B) imply that the constituents of the lyoprotectant hindered PBS salt formation, while some remaining nanoparticles were measured in the T 2% group. Crystalline salt particles were barely observed in the T 4% and TS 4% groups whose surface configurations appeared much smoother than those of the other groups (Fig. 4C and D). In particular, the TS 4% group demonstrated the most planar surface without any crystalline structures among all groups. The EDS elemental mapping results revealed that the smooth surfaces of the T 4% and TS 4% groups were covered with carbon (red) and oxygen (yellow). Two of the major atoms constitute the disaccharide lyoprotectant solutions (Fig. 4E and F, orange), while sodium and chlorine were also dispersed over the surfaces because their base solution was PBS. The glass transition temperature (Tg′) of the freeze-concentrated lyoprotectant solutions was examined by the differential scanning calorimetry (DSC) analysis with a heating program from −60 °C to +10 °C (Fig. 4G). The Tg′ of the TS 4% and 8% group was measured at −45.9 °C higher than those of the T 4% and TS 2% groups, both of which were measured at a similar level at −53.3 °C and −54.1 °C, respectively, while such glass transition process was not detected in the plot of the T 2% group.

**Fig. 5.**
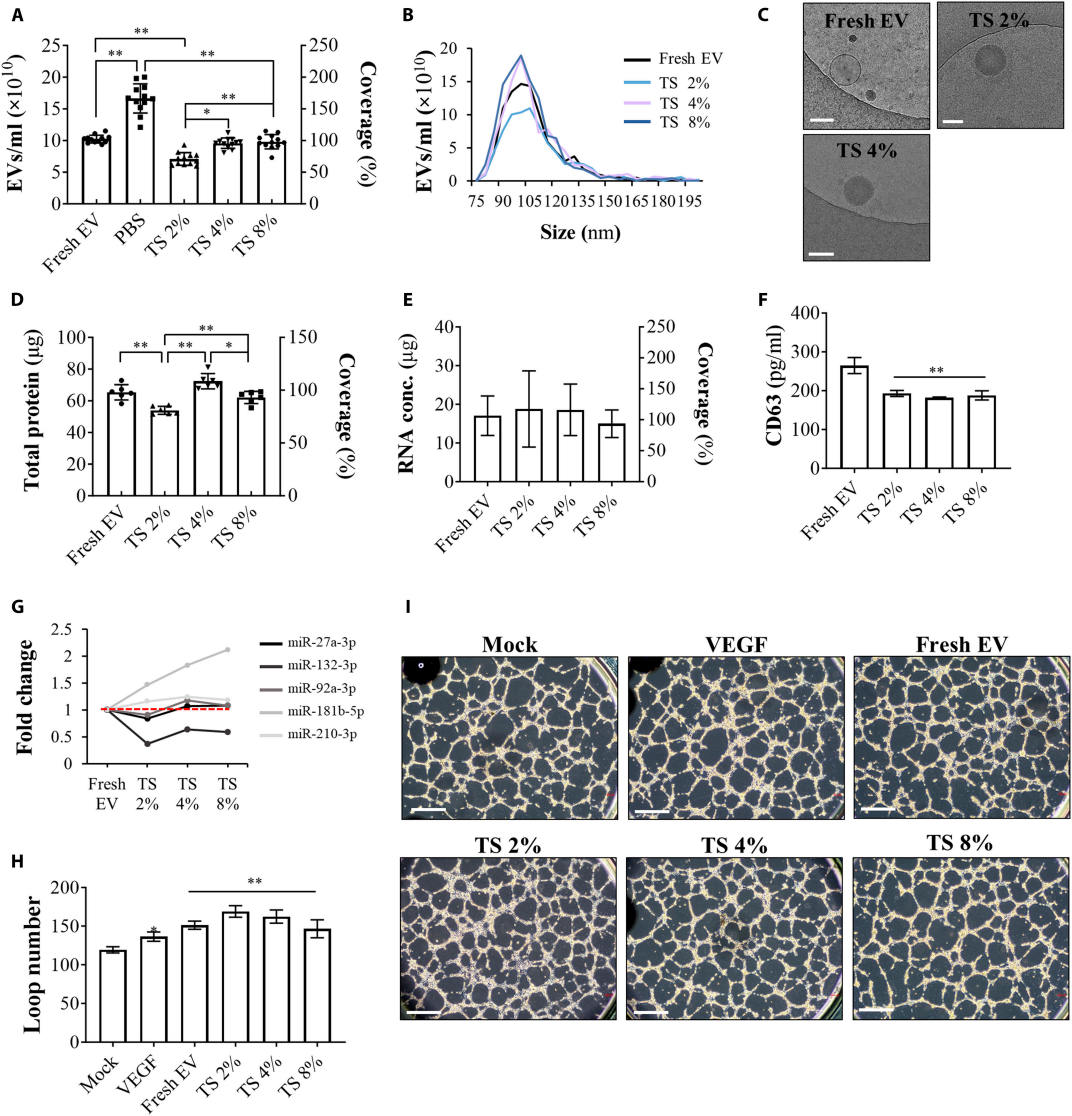
Evaluation of restoration capacity of EV characteristics after lyophilization. (A) Restoration of EV concentration lyophilized with PBS and TS 2%, 4%, and 8% compared to fresh EV (*n* = 12). (B) Number-based size distribution of fresh EV and lyophilized EVs with TS 2%, 4%, and 8%. (C) Cryo-transition electron microscopy (TEM) images of lyophilized EVs compared to fresh EVs. The scale bar indicates 100 nm. EV contents were investigated by (D) total protein detected using BCA protein assay (*n* = 8), (E) total RNA concentration (*n* = 6), (F) CD63 quantification conducted by ELISA (*n* = 3), and (G) fold change of miRNA levels calibrated with fresh EV (1; red line). The vascularization efficacy of lyophilized EVs was investigated using HUVEC tube formation assay. Each group was evaluated using (H) the number of loops (*n* = 5) and (I) the images of each group compared to the mock group. The scale bar indicates 200 μm. *P < 0.05 and **P < 0.001 were calculated by one-way ANOVA.

With convincing results showing that the mixture solutions of trehalose and sucrose successfully circumvented the PBS crystalline salt particle generation during the lyophilization (Fig. 4), EVs lyophilized with the TS 2%, 4%, and 8% were examined after rehydration for retained numbers, sizes, and morphologies of the EVs (Fig. 5A to C). Unlike the PBS group, the number of EVs in the TS 2%, 4%, and 8% groups did not increase from the initial numbers corresponding to that of the fresh EV group (Fig. 5A). In particular, the number of recovered EVs in the TS 4% and 8% group was comparable to that in the fresh EV group, while the TS 2% group displayed a lower recovery rate of EVs after rehydration. The rehydrated EVs in the TS groups showed size distributions similar to those of the fresh EV group, with well-preserved EV morphology (Fig. 5B and C). We also examined the total protein and RNA levels in the rehydrated EVs (Fig. 5D and E). The TS 4% and 8% group demonstrated a higher recovery rate of the total protein content than the TS 2% group, while no significant difference among all groups was found in the total RNA content, which retained levels similar to those of the fresh EV group. Our ELISA results showed that rehydrated EVs highly expressed CD63, one of the principal markers of EVs, although the degree of expression was lower than that in the fresh EV group (Fig. 5F). The analysis of miRNA expressions related to angiogenesis, such as miR-27a-3p, miR-132-3p, miR-92a-3p, miR-181b-5p, and miR-210-3p, revealed that the EVs reconstituted from the TS 2%, 4%, and 8% groups maintained the angiogenic capacity as therapeutic EVs, comparable to the levels of the freshly collected EVs (Fig. 5G) [25,26]. A HUVEC tube formation assay was conducted to examine the therapeutic ability of lyophilized and rehydrated EVs (Fig. 5H and I), which could be one of the prime indices to indicate angiogenic efficacy of the stem-cell-derived EVs. The degree of tube formation stimulated by the fresh EV, TS 2%, 4%, and 8% groups were quantitatively compared with a negative control (mock; basal medium) and positive control (VEGF; a conventional method for inducing tubular differentiation of HUVEC). The results demonstrated that the TS 2%, 4%, and 8% groups effectively stimulated HUVEC tube formation at levels comparable to those in the fresh EV- and VEGF-treated groups. Both TS 4% and 8% groups demonstrated superior capacity to maintain the native characteristics and therapeutic efficacy of EVs after lyophilization and rehydration. In our study, we chose the TS 4% group as the appropriate cryoprotectant composition while considering the cost-effectiveness as well as reducing the possible complexities that might arise from a dose control of rehydrated EV solution and its toxicity evaluation in the future GMP application.

**Fig. 6.**
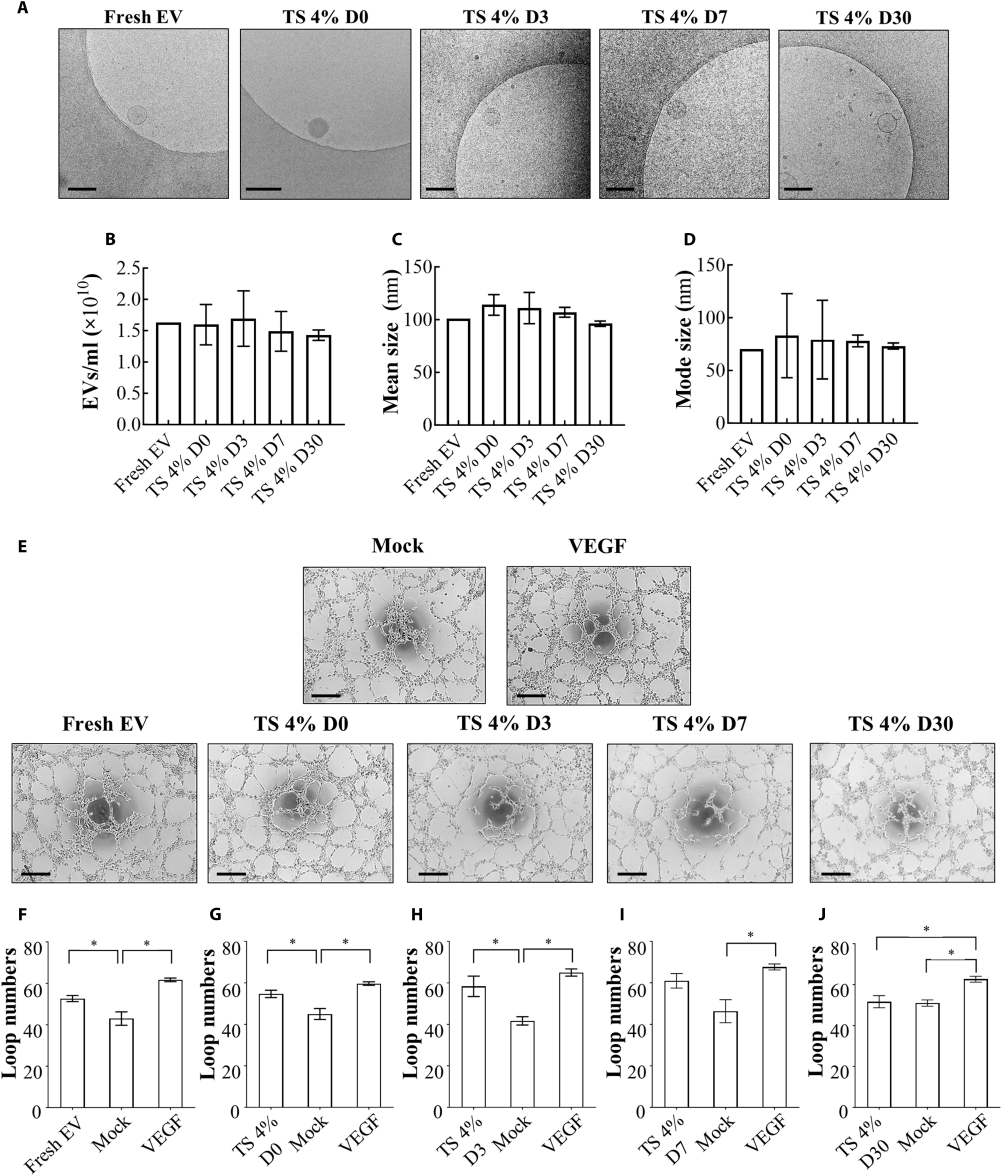
Evaluation of long-term preservation of EV characteristics derived from WJ-MSC lyophilized with TS 4%. The preservation of EV characteristics was evaluated by (A) Cryo-TEM images of lyophilized EVs at each post-lyophilization date, (B) EV concentration, (C) EV mean size, and (D) mode size (*n* = 5). The scale bar indicates 200 nm. Angiogenesis efficacy of EVs was investigated by (E) HUVEC tube formation assay and analyzed by (F to J) the loop number on each date (*n* = 5). The scale bar indicates 200 μm. *P < 0.05 was calculated by one-way ANOVA.

We also evaluated the lyophilized EVs from the TS 4% group in terms of their capacity for long-term preservation after 30 days of storage at room temperature. Concentrations of the rehydrated EVs were maintained at the initially stored level of the fresh EV group until day 30 with well-maintained membrane stability while preserving their size distributions similar to those of the fresh EVs (Fig. 6A to D). The angiogenic efficacy of EVs in the TS 4% group appeared to be maintained for up to 7 days of storage at the room temperature, whereas it was significantly diminished on day 30.

## Discussion

To preserve biological samples containing water, various lyoprotectants have been used to prevent physical and chemical damage and structural changes during freeze-drying [27–29]. Such reagents consist of cryoprotectant constituents and are often based on a buffer solution, such as PBS, to maintain ionic concentration and prevent damage from osmotic pressure to biological samples like EVs [24]. In this study, we attempted to lyophilize stem-cell-derived EVs using well-known cryoprotectant solutions such as DMSO, mannitol, trehalose, and sucrose.

The lyophilization of EVs using DMSO gave rise to heavily condensed slimy clumps, which required substantial effort to be completely rehydrated because of the collapse of cake, as previously reported [30]. In contrast, the EVs lyophilized in mannitol or trehalose solutions showed a typical lyophilized cake configuration, and after rehydration, the cakes appeared to dissolve in DI water immediately. However, our nanoparticle measurements showed unidentified nanoparticles with a similar size range to the EVs that were possibly generated during the lyophilization process and remained undissolved after rehydration. Our XRD and SEM/EDS analyses revealed that such undesired nanoparticles generated in all the rehydrated solutions were identified as PBS crystalline salts, staying undissolved for up to 48 h in DI water. Previous studies reported that lyophilization, particularly during drying, causes buffer salt crystallization in PBS-based solutions [31]. In the case of mannitol, although the samples were frozen at a temperature below Tg′, they can be crystallized due to their crystalline characteristics [32]. Mannitol was not used in this study since it forms mannitol β crystalline structures in addition to the PBS crystalline salts after lyophilization.

Trehalose possesses a high glass transition temperature and the ability to form an amorphous network with water effectively, providing strong amorphous characteristics that prevent water crystallization [33,34]. Due to these considerations, we used trehalose as the main lyoprotectant in our study. However, the 0.07 and 0.17% trehalose solutions in our experiments resulted in even higher PBS salt agglomeration compared to the PBS-only group. This result was found to align with previous reports that a low concentration of trehalose solution would rather bring about the accelerated buffer component crystallization in the lyophilization process because the scattered disaccharide molecules could serve as the basis for nucleation, where the formation of a crystal from a solution is initiated [35]. Therefore, we speculated that increasing the concentration of the trehalose solution would allow for higher amorphous stability during lyophilization and thus contribute to avoiding unwanted PBS crystalline salt formation [36,37]. Such a constant amorphous arrangement supported by an appropriate lyoprotectant could also play an important role in preventing physical and osmotic damage to biological samples, such as rupture of the EV’s lipid bilayer membrane, caused by locally concentrated hexagonal ice crystal formation, resulting in higher concentration gradients of solutes [38]. In addition, sucrose is also a non-crystalline disaccharide protectant like trehalose, frequently used for lyophilization. An amorphous reagent mixture of trehalose and sucrose as a lyoprotectant solution could sufficiently slow crystallization with affording relatively high Tg′, thus further contributing to the suppression of the PBS salt crystallization [39]. Our results demonstrate that the lyophilized cakes obtained from the TS 4% and 8% group were immediately dissolved after rehydration, without leaving any marks of PBS crystalline nanoparticles. SEM/EDS analyses revealed that any crystalline configurations were not observed in the TS 4% group, which displayed smooth planar surfaces covered with major atoms constituting the disaccharide solution.

Tg′ is one of the critical parameters for regulating amorphous phases of the lyoprotectant solution during freezing and drying processes [40,41]. The product temperature, which depends on the shelf temperature of the freeze-dryer, should be maintained below the Tg′ of the lyoprotectant solution to remain in the amorphous glassy state throughout the freezing and drying processes [42,43]. An appropriate lyoprotectant induces the formation of amorphous ice by reinforcing the amorphous phase of the solution to minimize the formation of hexagonal ice crystals when the water is frozen [44]. Our DSC analysis revealed that the Tg′ of the TS 4% and 8% group was increased by about 8 °C compared to the other groups. This increase is attributed to the added sucrose, which is known to prevent crystallization and elevate the glass transition temperature [39,45]. The results suggest that the TS 4% and 8% may reach the amorphous state more quickly than the other groups during the freezing process. If the temperature of the product during the drying process is higher than the Tg′ of the lyoprotectant, hexagonal ice particles may be recrystallized [46]. Thus, increasing the Tg′ of the solution can hinder the recurrence of ice nucleation and crystallization during the drying process. The increased Tg′ of the TS 4% group may contribute to the sample being sustained in the amorphous phase for a longer period during the drying process conducted at −50 °C, lower than the Tg′, thereby preventing recrystallization and reducing the formation of PBS crystalline nanoparticles.

After rehydration of the EVs lyophilized using TS 4% and 8%, most aspects of the generic characteristics of EVs, including number, size, morphology, protein and RNA content, and EV marker expression, were well maintained. Our miRNA expression analysis demonstrated that their therapeutic capacity was restored to the levels similar to those of freshly collected EVs. With having similar EV preservation capacities after lyophilization and rehydration between the TS 4% and 8% groups, we conducted long-term preservation experiments using the TS 4% group. In long-term preservation at room temperature, EVs lyophilized in the TS 4% group sustained their morphologies, numbers, and sizes for up to 30 days, although their therapeutic capacity was likely weakened after 7 days. Maintaining moisture contents as low as possible after lyophilization is crucial to sustaining long-term stability of the lyophilized cakes; otherwise, the cakes could be collapsed, degraded, as well as lose their own biological potency [47]. Adjusting a secondary drying process during lyophilization could reduce the residual moisture in the lyophilized cakes. However, some studies have identified potential drawbacks of excessive drying, including the disruption of hydrogen bonds between proteins and water, leading to a structural change and decrease in product stability [48,49]. Therefore, further research on the effective method to control the residual moisture in the lyophilized biological samples is required. In addition, vacuum packaging or the use of rubber stoppers for the lyophilized cakes could also alleviate the concerns of sample re-moisturization during long-term storage [50].

## Conclusion

The freeze-dried formulation of stem-cell-derived EVs can be applied to a wide range of clinical cases while facilitating adaptable multiple dose controls in real time at the bedside, as well as broadening the possible treatment routes in addition to systemic administration, such as oral administration, nasal inhalation, and external skin preparations [51–53]. However, the PBS crystalline nanoparticles generated in the lyophilization process and remaining undissolved after rehydration can cause significant distractions in the measurement of EV concentrations, thus leading to complications in validating accurate dose control and investigating the therapeutic effects and biological responses of EV treatment [54,55]. Moreover, such errors in the EV concentration measurement could also be problematic in setting a rational quality control standard for EV therapeutics in good manufacturing practice because their identity, purity, and potency could vary depending on the misled EV concentrations. We believe that our study provides meaningful information on the appropriate composition of lyoprotectants, particularly for EV lyophilization, encouraging the applications of EV therapeutics in the health industry.

## Data Availability

The data that support the findings of this study are available from S&E bio, Co., Ltd. but restrictions apply to the availability of these data as a patent application has been submitted, and so are not publicly available. Data are however available from the corresponding author upon reasonable request and with permission of S&E bio, Co., Ltd.
